# Patches and Blebs: A Comparative Study of the Composition and Biophysical Properties of Two Plasma Membrane Preparations from CHO Cells

**DOI:** 10.3390/ijms21072643

**Published:** 2020-04-10

**Authors:** Bingen G. Monasterio, Noemi Jiménez-Rojo, Aritz B. García-Arribas, Howard Riezman, Félix M. Goñi, Alicia Alonso

**Affiliations:** 1 Instituto Biofisika (CSIC, UPV/EHU), Campus Universitario, 48940 Leioa, Spain; bingen_gm@hotmail.com (B.G.M.); aritzgarciaar@hotmail.com (A.B.G.-A.); felix.goni@ehu.es (F.M.G.); 2 Departamento de Bioquímica, Universidad del País Vasco, 48940 Leioa, Spain; 3 NCCR Chemical Biology, Department of Biochemistry, University of Geneva, 1211 Geneva, Switzerland; Noemi.Jimenez@unige.ch (N.J.-R.); Howard.Riezman@unige.ch (H.R.)

**Keywords:** giant plasma membrane vesicles, blebs, plasma membrane, general polarization, Atomic Force Microscopy (AFM), lipidomics

## Abstract

This study was aimed at preparing and characterizing plasma membranes (PM) from Chinese Hamster Ovary (CHO) cells. Two methods of PM preparation were applied, one based on adhering cells to a poly-lysine-coated surface, followed by hypotonic lysis and removal of intracellular components, so that PM patches remain adhered to each other, and a second one consisting of bleb induction in cells, followed by separation of giant plasma membrane vesicles (GPMV). Both methods gave rise to PM in sufficient amounts to allow biophysical and biochemical characterization. Laurdan generalized polarization was used to measure molecular order in membranes, PM preparations were clearly more ordered than the average cell membranes (GP ≈0.450 vs. ≈0.20 respectively). Atomic force microscopy was used in the force spectroscopy mode to measure breakthrough forces of PM, both PM preparations provided values in the 4–6 nN range, while the corresponding value for whole cell lipid extracts was ≈2 nN. Lipidomic analysis of the PM preparations revealed that, as compared to the average cell membranes, PM were enriched in phospholipids containing 30–32 C atoms in their acyl chains but were relatively poor in those containing 34–40 C atoms. PM contained more saturated and less polyunsaturated fatty acids than the average cell membranes. Blebs (GPMV) and patches were very similar in their lipid composition, except that blebs contained four-fold the amount of cholesterol of patches (≈23 vs. ≈6 mol% total membrane lipids) while the average cell lipids contained 3 mol%. The differences in lipid composition are in agreement with the observed variations in physical properties between PM and whole cell membranes.

## 1. Introduction

Model membranes have been widely used in membrane biophysical studies. Among many others, they have made possible the observation of heterogeneities in synthetic lipid membranes [1]. However, even if model membranes are good tools for the reconstitution of molecular or cellular systems and for mimicking many of their properties, the native cell membrane organization remains unclear. Model membranes do not maintain the compositional complexity and the ability for protein synthesis, they lack the complex protein–lipid interactions found in nature, and the cytoskeleton is not present [2,3]. Taking this into account, subcellular membrane preparations are now more than ever required for biochemical and biophysical studies. The plasma membrane (PM), due to its particular role as the interface between the cell and its environment, is the object of special attention from molecular and cell biologists.

It is then unfortunate that most of the available PM preparation methods are time-consuming and often give rise to samples in poor yields and/or contaminated with other cell membranous fractions. Most current methods for PM isolation are based on serial steps of differential centrifugation [4]. Attempts to achieve a better PM purification yield include the use of magnetic beads with immobilized monoclonal antibodies against specific membrane proteins [5]. Moreover, it has been proved that differently coated superparamagnetic iron oxide nanoparticles (SPIONS) have the ability to reside at specific cell compartments. Aminolipid coated SPIONS for example, can reside at the PM allowing its magnetic purification [6]. Surface-biotinylation is another useful strategy; after the cell surface is biotinylated, a competitive biotin elution strategy can be employed for PM isolation [7]. Still, other methods are based on the use of poly-lysine-coated acrylamide beads that bind PM in cell lysates [8]. An additional PM isolation technique is lectin-affinity chromatography [9]. This method takes advantage of the specific membrane sugars. Lectins are a group of carbohydrate-binding proteins with different sugar-binding specificities. In the PM the sugar residues are always exposed on the outside leaflet, while in intracellular membranes they are located towards the inner organelle compartment. Among lectins, concanavalin A is most frequently used for the binding of glycosylated membrane proteins [9,10].

The present study is focused on describing the composition and physical properties of PM from Chinese Hamster Ovary (CHO) cells. Since the first CHO line was derived, around 1957, CHO cells have been a cell line of choice because of their rapid growth in suspension culture and high protein production. In order to reinforce the value of our observations, PM obtained using two very different preparation methods were used. One of them was developed by Bezrukov et al. [11] to isolate large quantities of directly accessible PM from attached cells. The method is based on the adhesion of cells to a poly-lysine coated surface followed by hypotonic lysis with ice-cold distilled water and subsequent washing steps to achieve intracellular content elimination. The resulting ‘patches’ happen to be a very good PM preparation. The second method was based on the preparation of giant plasma membrane vesicles (GPMV, or blebs), that are also considered to be isolated PM samples [12,13]. These GPMV can be formed through addition of 1–5% (*v*/*v*) ethanol, acetone, formaldehyde or DMSO to cells in culture [13]. A hypertonic vesiculation buffer containing chloride salts can also be employed for the same purpose [14]. These ‘blebs’ can be conveniently observed using optical microscopy.

Microscopic observations, Laurdan fluorescence general polarization (GP) and atomic force microscopy (AFM) have been used in combination with mass spectrometric analysis to characterize and compare the ‘bleb’ and ‘patch’ PM preparations. Laurdan GP and AFM force spectroscopy have been selected because, having very different physical foundations, they both report on related parameters, molecular membrane order and membrane mechanical resistance to penetration by a microprobe. Mass spectrometry provides information on a chemical parameter, namely lipid composition of the membrane, that would constitute the material substrate for the observed physical properties.

## 2. Results and Discussion

### 2.1. PM Purification

**Figure 1:**
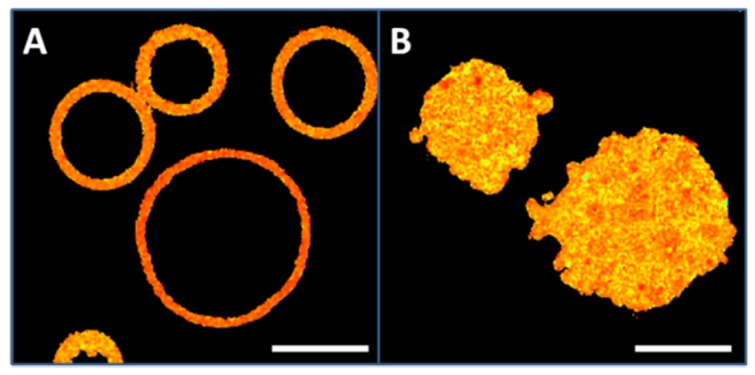
Plasma membrane preparations from CHO cells. (**A**), giant plasma membrane vesicles (GPMV) or blebs. (**B**), PM patches. Bar 10 μm.

Fluorescently labeled CHO cell PM patches and GPMV (blebs) were visualized by confocal microscopy. Figure 1A shows a number of GPMV labeled with Laurdan. GPMV have a spherical shape and, as they do not contain any internal structures, they adopt a ring morphology in their equatorial plane images. At variance, PM patches are adhered to a surface and they exhibit a planar structure. Thus, they appear labeled all along their surface (Figure 1B). According to their sizes, GPMV diameters vary between 3 and 20 µm (FWHM: 12.3 ± 3.3, mean value ± SD) while PM patches have diameters between 8 and 25 µm (FWHM: 16.6 ± 4.4, mean value ± SD).

### 2.2. Laurdan Fluorescence GP

**Figure 2:**
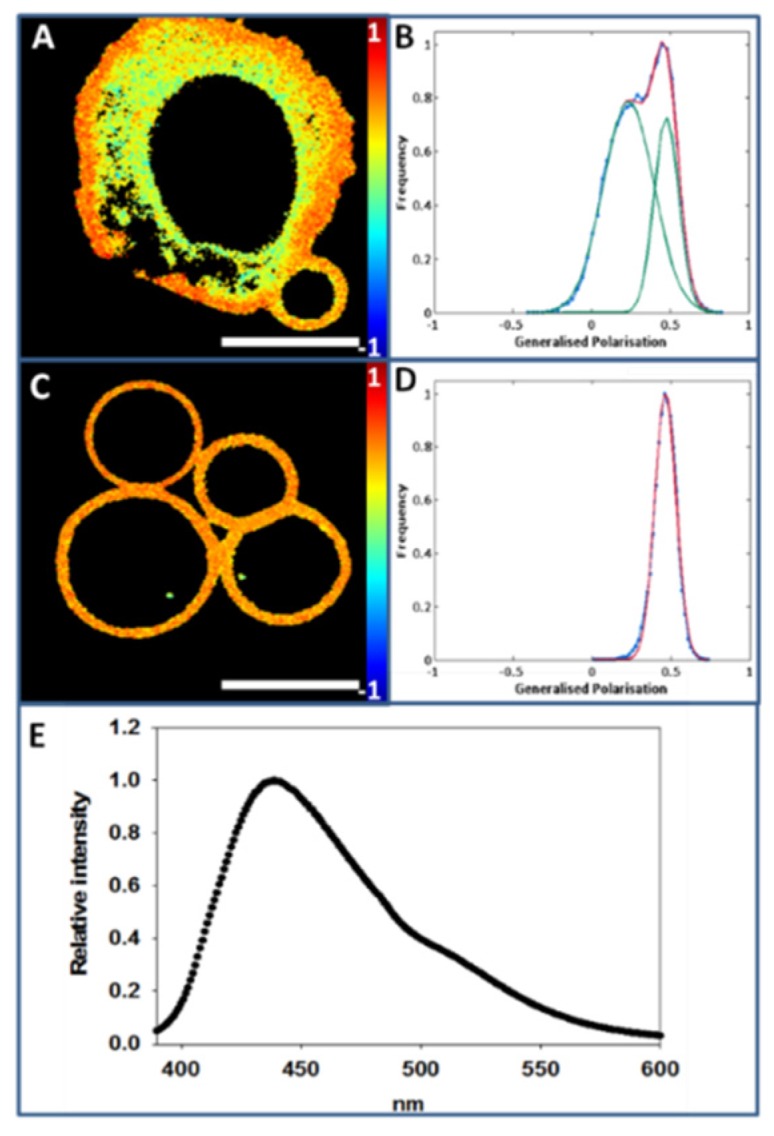
GPMV (bleb) formation and Laurdan GP measurements at 20 °C. (**A**), GPMV formation from a CHO cell (average GP value 0.44). (**B**), Generalized polarization plot of image A. (**C**), Laurdan staining of GPMV derived from CHO cells. (**D**), Generalized polarization plot of image C. (**E**), Laurdan emission spectrum of CHO cell blebs. Bar 10 µm.

In Figure 2A paraformaldehyde- and dithiothreitol-mediated GPMV formation in a CHO cell can be observed. Two different regions in Laurdan-labeled bleb-forming cell images can be distinguished, a more rigid region (orange-red) that corresponds to the PM, and a more fluid region (yellow-green) that corresponds to the intracellular area. GP analysis shows that, in a −1 to +1 scale, PM GP value is 0.52 ± 0.04 (at 20 °C), while the corresponding intracellular value is 0.20 ± 0.08 (at 20 °C) (Figure 2B). A number of control experiments were performed in order to strengthen the above data. GP measurements in Figure 2 were made for cells grown on a glass surface, while in other experiments the PM fractions are examined on polylysine-coated glass. Thus the following control measurements were performed with cells (at 20 °C): (i) glass without polylysine (GP: 0.520 ± 0.02), vs. glass with polylysine (GP: 0.516 ± 0.02), no significant differences were detected; (ii) cells treated with PFA and DTT, no polylysine, (GP: 0.473 ± 0.03), a slight but significant (*p* < 0.001) decrease with respect to untreated cells (0.52 ± 0.04) was measured.

After GPMV (bleb) formation, whole cells were removed from the medium, and isolated GPMV images were taken (Figure 2C). Color intensity analysis revealed a homogenous population with an average GP value 0.47 ± 0.06 (at 20 °C) (Figure 2D). Figure 2E shows the emission spectrum of isolated GPMV. The spectrometric analysis performed at a constant temperature of 20 °C results in a maximum peak around 440 nm, suggesting that under these conditions the GPMV surface exhibits on average the properties of an ordered phase. This is compatible with the coexistence of an overall ordered phase with more fluid (and/or even more rigid) nanodomains that cannot be resolved with the available technology.

**Figure 3:**
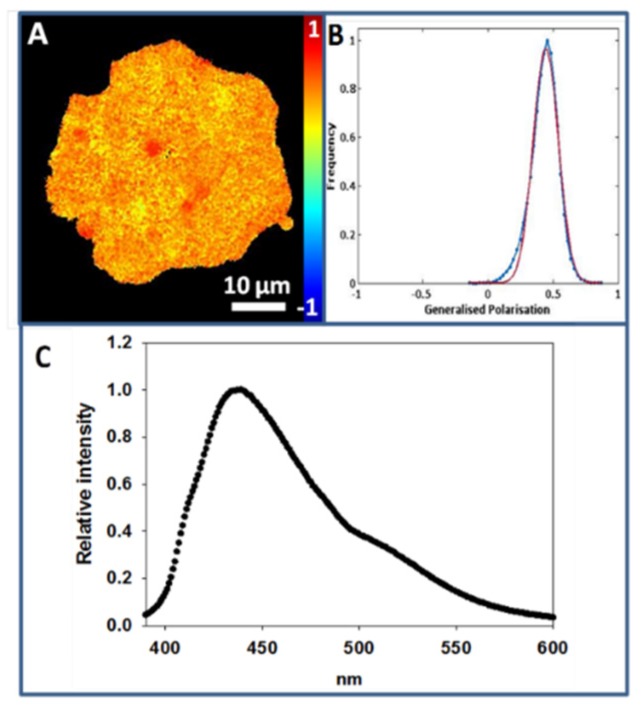
Laurdan staining of plasma membrane patches (at 20 °C). (**A**), Laurdan staining of a CHO cell plasma membrane patch. (**B**), Generalized polarization plot of image A. (**C**) Laurdan emission spectrum of CHO cell PM patches.

The PM purification process makes patches expand, causing them to have an irregular morphology. In Figure 3A an individual Laurdan-labeled CHO cell PM patch is displayed. The color intensity graph associated with GP analysis throughout the patch detects a single peak around 0.44 ± 0.05 (at 20 °C) (Figure 3B).

Figure 3C corresponds to the emission spectrum of Laurdan in PM patches as observed in a spectrofluorometer. After scraping the surface-adhered patches, Laurdan-labeled patch suspensions were spectrofluorometrically analyzed at a constant temperature of 20 °C, and a peak with a maximum emission intensity around 440 nm was measured, indicating that most of the lipid acyl chains in the PM patches are in an ordered state under these conditions. The same observation was made on GPMV, and again this does not rule out the presence of nanodomains with different degrees of molecular order in the patches. 

**Figure 4:**
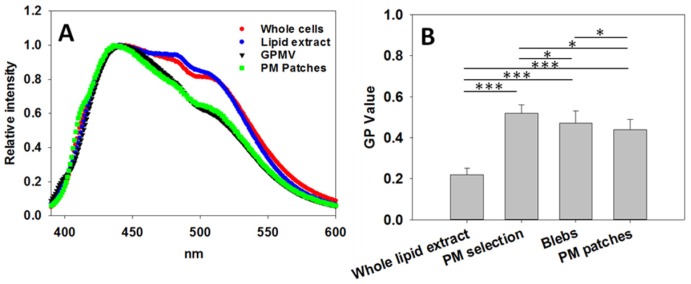
Laurdan GP measurements. (**A**), Laurdan fluorescence emission spectra. Red, whole CHO cells; blue, SUV formed from CHO cell lipid extract; black, GPMV (blebs) from CHO cells; green, PM patches from CHO cells. Spectra retrieved at 40 °C. (**B**), Laurdan GP values obtained from microscopy images such as shown in Figure 1, 2A, 2C, 3A, (at 20 °C, *n* = 150, value = mean + SD). Statistically significant differences were calculated with ANOVA and Student’s t-test. Significance: (*) *p* < 0.05; (***) *p* < 0.001.

In Figure 4 Laurdan GP spectra from the various samples at 40 °C can be compared. Whole cells and SUV formed from whole cell lipid extracts exhibit a similar behavior (Figure 4A). In turn GPMV and PM patches also give rise to virtually superimposable spectra. The latter spectra are shifted to lower wavelengths indicating that the PM samples have a more ordered structure than the membranes from whole cells or from whole cell lipid extracts (Figure 4A). These results are in accordance with previous studies [15,16,17]. For a further confirmation of this issue, Laurdan emission spectra were measured at 20 °C and 30 °C, with essentially similar results (Figure S1).

Figure 4B underlines the existing differences in GP between PM preparations, whole cells and lipid extract. Data were extracted from microscopy images (at 20 °C) as shown in Figure 2, 3. Statistically (ANOVA and Student’s t-test), very significant differences exist between the whole cell lipid extracts and the PM measurements (PM selection from whole cell pictures, blebs and PM patches). Lipid extract GP value is lower than all PM measurements, meaning that the extract gives rise to bilayers less ordered than the PM. This suggests that, taking into account the ensemble of membranes in a cell, and according to lipid order criteria, two different populations exist, namely PM and intracellular membranes. It is known that PM is more ordered than intracellular membranes [15]. In the whole-cell lipid extract, intracellular membranes will have a much higher relative weight than PM, and the GP value of the latter will hardly be affecting the overall GP value. This results in a lipid extract GP value closer to the value of intracellular membranes, specifically 0.22 ± 0.03 (at 20 °C).

There are also small differences when the various PM preparations are compared (Figure 4B). The GP value of PM selection is 0.52 ± 0.04, GP of GPMV is 0.47 ± 0.06, and that of the PM patches is 0.44 ± 0.05 (at 20 °C). The differences, according to the ANOVA and Student’s t-test, are considered to be statistically very significant, perhaps revealing differences between the various methods of PM preparation.

### 2.3. AFM Microscopy and Force Spectroscopy

**Figure 5:**
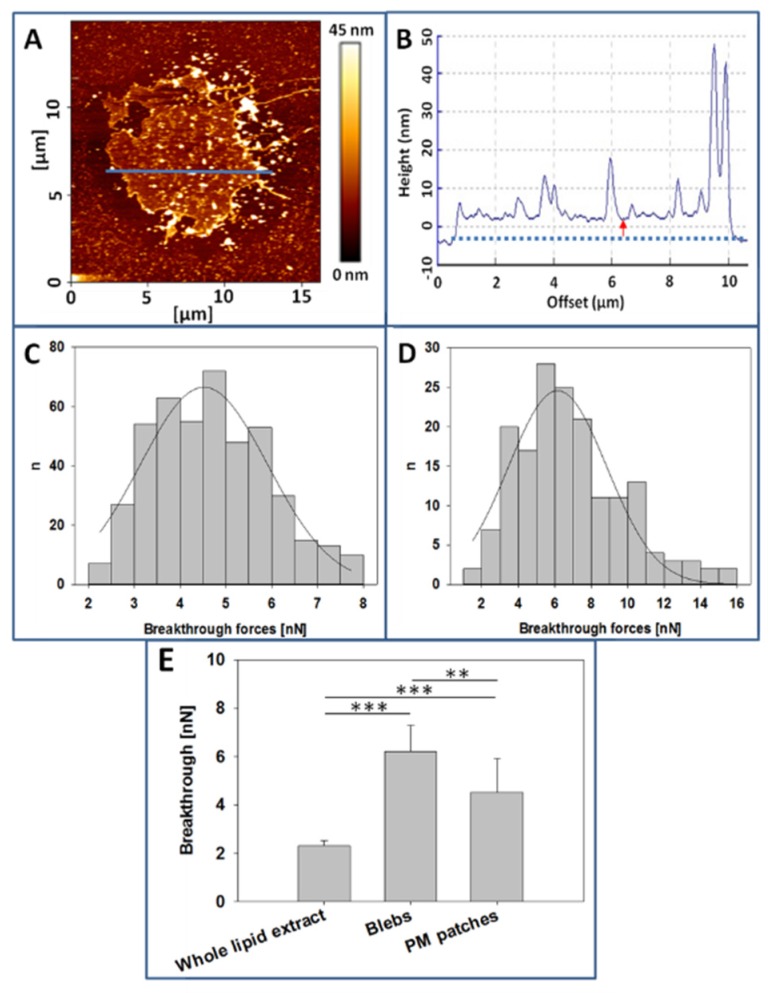
Atomic Force Microscopy (AFM) measurements. (**A**), CHO cell plasma membrane patch over polylysine-coated mica. (**B**), Topographic image of the cross-section indicated by the blue line in 5A. (**C**), Breakthrough forces distribution of CHO cell plasma membranes (PM) patches. (**D**). Breakthrough forces distribution of CHO cell blebs. (**E**). Comparison of breakthrough forces, data extracted from experiments as in C, D. Whole lipid extract breakthrough value was obtained from supported planar bilayers formed with CHO cell lipid extracts. (*n*= at least 160, value = mean + SD) Statistically significant differences were calculated with ANOVA and Student´s t-test. Significance: (**) *p* < 0.01; (***) *p* < 0.001.

Figure 5A shows the AFM topographic image of a CHO cell PM patch. This patch has irregular borders and an average diameter of 10 µm. In Figure 5B we can see the cross-section corresponding to the blue line in Figure 5A. The red arrow corresponds to the PM patch average thickness (≈4 nm), in good agreement with the commonly observed thickness of fluid lipid bilayers. There are also localized regions with much higher thicknesses, perhaps due to aggregates of proteins, sugars, or perhaps folds formed in the patch isolation process. Figure 5C depicts the distribution of breakthrough forces in the PM patches, with a mean value of 4.5 ± 1.4 nanonewtons (nN), while in Figure 5D we can observe the breakthrough forces distribution of CHO cells GPMV with a mean value of 6.2 ± 1.1 nN. GPMVs topographic images could not be retrieved because of difficulties to achieve a good GPMV membrane extension on the mica surface.

Regarding the mechanical resistance of bilayers, as measured by AFM in the force spectroscopy mode, breakthrough forces of CHO cell lipid extracts are on average 2.3 ± 0.22 nN, those of GPMV are of 6.2 ± 1.1 nN, and those corresponding to PM patches are of 4.5 ± 1.4 nN (Figure 5E). The observed differences are statistically significant, as detailed in the figure. As occurs with GP values, whole cell lipid extract breakthrough forces are smaller than those of PM preparations (see the correlation between Laurdan GP values and AFM breakthrough forces in Figure S2). 

The differences between GPMV (blebs) and patch breakthrough forces are not easy to explain. Changes induced by paraformaldehyde during GPMV preparation may be an important factor, and it should be noted that the same reagent is commonly used in cell and tissue fixation protocols. 

### 2.4. Lipidomic Studies

**Figure 6:**
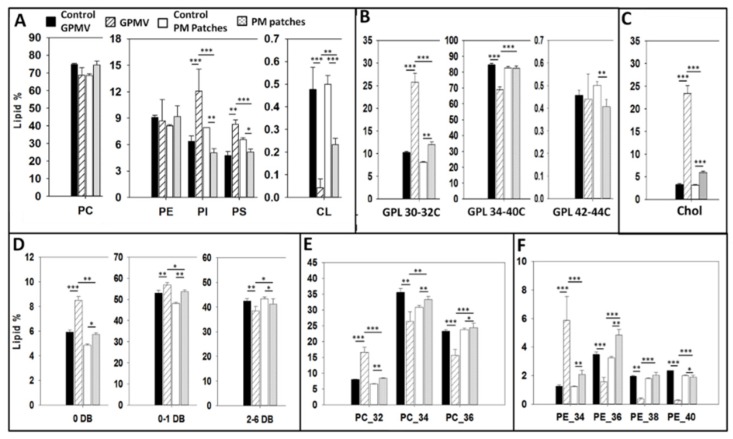
Mass spectroscopy lipidomic analysis of whole cells and plasma membrane preparations. (**A**) Total phospholipids; (**B**) Short, long and very long glycerophospholipids; (**C**) Total cholesterol; (**D**) Phospholipid saturation level (DB = double bond); (**E**) Phosphatidylcholine distribution according to chain length; (**F**) Phosphatidylethanolamine distribution according to chain length. Bras: solid black, whole cells treated for GPMV preparation; striped, GPMV (blebs); empty, cells treated for PM patch preparation; dotted PM patches. Significance: (*) *p* < 0.0 (**); *p* < 0.01; (***) *p* < 0.001.

The lipidomic data of the various membrane preparations under study are shown in full in the Supplementary Material (Table S1 and Figure S3). A summary of the lipid distributions of whole CHO cells and different PM preparations is shown Figure 6. Figure 6A shows the percent distribution of phospholipids, of which phosphatidylcholine constitutes about 70% of the total phospholipid in all measured samples. With respect to phosphatidylethanolamine (≈10%), a molecule known to help maintain the membrane fluidity homeostasis together with cholesterol and the GPL saturation degree [18], there is no significant difference either between whole cells and PM preparations (Figure 6A). The PE/PC ratio has been proposed as a useful parameter that influences membrane fluidity [18]. From the lipidomic data we have computed PE/PC ratios of 0.121 and 0.126, i.e. virtually identical, respectively for control cells and GPMV. The corresponding values for whole cells and PM patches were also identical, 0.134 and 0.136. Regarding phosphatidylinositol and phosphatidylserine, statistically significant differences exist between whole cell and PM preparations. These phospholipids appear in smaller amounts in PM patches (but not in GPMV) than in whole cells. PI seems to follow the behavior observed by Kalvodova et al. [19] with baby hamster kidney (BHK) cells and by Lorizate et al. with HeLa and MT4 cells [20]. Cardiolipin, a lipid that constitutes 10% of phospholipids in mitochondrial membranes [21] does not make up more than 0.5% of the total phospholipid in any of our samples (Figure 6A). 

Glycerophospholipid acyl chain length distribution in whole CHO cells (Figure 6B) is similar to the one seen by Sampaio et al. [22] and by Gerl et al. [23] with Madin-Darby canine kidney (MDCK) cells. Regarding PM preparations (Figure 6B) short-chain (30–32C) glycerophospholipids are more abundant in GPMV (blebs) and PM patches than in whole lipid extracts. This short-chain glycerophospholipid increase is compensated by a lower proportion of long-chain glycerophospholipids in PM preparations. ANOVA and Student´s t test shows that differences are statistically significant. Among PM samples, GPMV have more short- and less long-chain (34–40C) glycerophospholipids than PM patches. The very long-chain (42–44C) glycerophospholipids exist in very small proportions and exhibit small variations among the different preparations.

Lipid unsaturation data are summarized in Figure 6C. Whole CHO cell unsaturation values are similar to the ones observed in previous studies [21]. Statistically significant differences exist if we compare whole cell lipid extracts and PM preparations; the latter contain less polyunsaturated and more saturated phospholipids.

Comparing the CHO phosphatidylcholine species according to their fatty acid chain length (combined chain lengths of both fatty acyl residues) (Figure 6E), the 32C species i.e. those PC containing 32 C atoms in the sum of both acyl chains, are more abundant in PM preparations, and the same is true of the 34C and 36C ones in patches, but not in blebs (Figure 6E). This result is in accordance with the one obtained by Lorizate et al. [20] who observed that 32C PC are increased in HeLa and MT4 cells PM. With respect to the phosphatidylethanolamine fatty acid composition, the most relevant observation is that 40C phosphatidylethanolamines are less abundant in any PM preparations than in whole cells (Figure 6F). In addition, 34C are significantly more abundant in both PM patches and GPMV, as observed by Lorizate et al. [20] with HeLa cells. Considering 36C and 38C PE, statistically significant differences exist between GPMV (blebs) and PM patches, in principle because of the different preparation techniques.

Whole cell extracts have lower cholesterol amounts than PM preparations. These results are in accordance with previous studies [7,19,20,24] showing that PM is enriched in cholesterol. Sezgin et al. have proved that MβCD-mediated cholesterol depletion decreases PM GP values. This means that cholesterol, together with saturated GPL confers more rigidity to the PM as compared with intracellular organelles [25]. Only 3.3 ± 0.2% of total lipid is cholesterol in the whole cell lipid extract. Nevertheless, this value is increased in PM preparations: up to 5.9 ± 0.3% in PM patches and up to 23.4 ± 1.77% in GPMV (Figure 6C). The higher cholesterol concentrations in PM, together with the lower proportions of polyunsaturated fatty acids (Figure 6D) are in agreement with the Laurdan (Figure 4) and AFM (Figure 5) results showing that PM is more ordered and packed than the whole cell lipid extract. We do not have at present an adequate explanation for the difference in cholesterol contents between GPMV and patches. Similar high cholesterol contents have been found in other plasma membrane preparations, GPMV or silica bead methods [20]. However, this might be related to the observation by Keller et al. [26] that lateral lipid separation occurs in the plasma membrane, at least during the procedure of GPMV preparation.

## 3. Materials and Methods

### 3.1. Cell Growth

The wild type CHO-K1 (ATCC CCL-61, from ATCC, Manassas, VA, USA) cell line has been used to perform this study. Cells were grown in DMEM:F12 (Dulbecco’s Modified Eagle *Medium*: Nutrient Mixture *F-12*) medium, containing 10% FBS (Fetal Bovine Serum), 100 U/mL penicillin, 100 U/mL streptomycin, and 6 mM Gln (GlutaMax supplemented) at 37 °C and 5% CO_2_ humidified atmosphere. All cell culture products were purchased from Thermofisher (Waltham, MA, USA).

### 3.2. Sample Preparation

Intact cells, whole-cell lipid extracts, GPMV or blebs, surface-attached PM patches, and giant unilamellar vesicles (GUV) were used to perform the measurements.

#### 3.2.1. Cell Lipid Extraction

Lipid extraction was performed as in Ahyayauch et al. [27]. Briefly, cell pellets were first resuspended in perchloric acid (60% *v*/*v*), samples were then centrifuged at 14,000g for 15 min and supernatants were discarded. Pellets were moved to an extraction tube, resuspended in 2.5 mL chloroform: methanol (2:1) (*v*/*v*) and mixed for 15 min. Then 5 mL cold 0.1 mM HCl solution was added to the mixtures. After homogenizing, samples were centrifuged at 1700g for 20 min. Supernatants were discarded, while the lipid-containing organic phases stayed in the lower phase. Phospholipid concentrations were determined by phosphate analysis.

#### 3.2.2. GPMV (bleb) Formation

CHO cell blebbing was induced to perform GPMV formation. Cells were grown to confluence as described above in T25 flasks and washed twice with GPMV buffer (2 mM CaCl_2_, 10 mM HEPES, 150 mM NaCl, pH 7.4). Cells were then incubated with GPMV formation reagent (freshly prepared 2 mM dithiothreitol and 25 mM paraformaldehyde in GPMV buffer) for 1 h at 37 °C. After incubation, the GPMV-containing GPMV reagent was collected from the flasks and centrifuged at 14,000g for 20 min. Supernatant was discarded. Several washing and centrifugation steps were conducted to eliminate dithiothreitol and paraformaldehyde traces. Finally, GPMV were resuspended in 500 µL GPMV buffer [28].

#### 3.2.3. Isolation of PM Patches

Cell PM was isolated essentially as described by Bezrukov et al. [11]. In summary, glass bottom dishes or mica slips were covered with poly-lysine and left in the laminar flow cabinet for 30 min at room temperature under UV radiation. Then excess poly-lysine was removed, and dishes or mica slips washed twice with PBS. Cells were seeded and grown to 50% confluence and incubated for 2 h for adherence to the support. After incubation, two washing steps were performed using cold Tris saline buffer (150 mM NaCl, 25 mM Tris, 2 mM KCl, pH 7.4) to discard non-attached cells. Then cells were allowed to swell in cold distilled water for 2 min. Mechanical cell disruption was achieved with a pressure stream formed by a 20-mL syringe coupled to a 19X1-1/2(TW)A needle. For this purpose, dishes were oriented at 60 deg to the stream and slightly rotated during the process. As a result, intracellular contents were released while PM stayed attached to the support. Finally, several washing steps were performed to discard remaining intracellular contents.

In order to check the purification quality, intact cells and purified PM patches were stained using Di-4-ANEPPDHQ (λ_ex_ = 465 nm, λ_em_ = 635 nm) as a general fluorescent stain, together with organelle-specific fluorophores. Nuclei were stained using 2.8 µM Hoechst 33342 (λ_ex_ = 361 nm, λ_em_ = 497 nm) for 10 min at 37 °C, Golgi apparatus was stained using 10 µM BODYPY FL C5-ceramide (λ_ex_ = 500 nm, λ_em_ = 510 nm) for 30 min at 37 °C, and mitochondrial staining was performed with 0.75 µM Mitotracker Green (λ_ex_ = 488 nm, λ_em_ = 510 nm) for 30 min at 37 °C. All fluorophores were purchased from Thermofisher (Waltham, MA, USA).

Images were visualized in a Leica TCS SP5 II confocal microscope (Leica Microsystems GmbH, Wetzlar, Germany) at room temperature and ImageJ software was used in order to measure fluorescent intensity. The fluorescence intensity of each marker was comparatively measured in PM patches and intact cells, and specific organelle contamination was quantified. The results are shown in Supplementary Figure S4. In Figure S4A,D we can observe intact cells and PM patches stained with the general stain Di-4 ANEPPDHQ and the nuclear stain Hoechst 33342; only 1.4% of the nuclei remain in the PM patches sample. In Figure S4B we can see Mitotracker Green-mediated mitochondrial staining. After PM purification, the mitochondrial Mitotracker signal is of 4.7% of the original (Figure S4B,E). Figure S4C shows Golgi staining by Bodipy-FL-C%-Cer fluorophore. Figure S4F shows that 6.8% of Golgi membranes remain in the sample after osmotic shock-mediated PM formation. The numerical data allow a comparison of our degree of purity with that obtained by Bezrukov et al. [11]. Both sets of data are very similar.

#### 3.2.4. GUV Formation

GUV were formed in a PRETGUV 4 chamber supplied by Industrias Técnicas ITC (Bilbao, Spain) using the modified electroformation method first developed by Angelova and Dimitrov [29].

#### 3.2.5. SUV Formation

The lipid extracts were evaporated under N_2_ gas, kept under vacuum for 2 h to remove solvent traces, and the lipids were swollen in PBS buffer. SUV were obtained by sonicating the swollen lipid suspensions with a probe-type Soniprep 150 sonicator (MSK, London, UK).

### 3.3. Laurdan General Polarization

Laurdan fluorescence GP is often used as an indication of membrane fluidity/rigidity. When a lipid membrane is in the gel phase Laurdan emission peaks at 440 nm, whereas in the liquid crystalline phase the spectrum is red-shifted to approximately 490 nm [30]. GP measurements were conducted in either intact cells, GPMV, PM patches or model membranes (SUV or GUV) formed from lipid extracts.

#### 3.3.1. Intact Cells

Cells grown in glass bottom dishes as previously described were stained with 5 µM (final concentration) Laurdan (Molecular Probes, Eugene, OR, USA) for 5 min, several PBS washing steps were performed, and cells were left in this buffer for visualization.

#### 3.3.2. Blebs (GPMV)

Blebs were mixed with 5 µM Laurdan, added to poly-lysine-coated glass-bottom dishes (MatTek, Ashland, OR, USA) and left for 3 h in order to sediment before visualization.

#### 3.3.3. Patches

Poly-lysine-coated glass-bottom dishes were used to form PM patches. Patches were stained with Laurdan at a final concentration of 5 µM for 5 min, several PBS washing steps were performed, and cells were left in PBS for visualization.

#### 3.3.4. GUV

0.2 mM lipid extract in chloroform:methanol (2:1, *v*/*v*) was mixed with 0.01 mM Laurdan. 3 µL of this lipid stock was deposited onto the surface of Pt electrodes and solvent traces were removed under high vacuum for at least 2 h.

The Pt electrodes were then covered with 400 μL 300 mM sucrose buffer and the Pt wires were connected to an electric wave generator (TG330 function generator, Thurlby Thandar Instruments, Huntington, UK) under alternating current field conditions (10 Hz, 2.5 VRMS for 120 min) at 37 °C. After GUV formation, the chamber was placed on an inverted confocal fluorescence microscope for GUV visualization. 

### 3.4. Image Acquisition

Images were acquired on a Leica TCS SP5 II microscope (Leica Microsystems GmbH, Wetzlar, Germany). Samples were imaged through a 63 × water-immersion objective (numerical aperture, NA = 1.2), and 512 × 512 pixels images were acquired at 400 Hz per scanning line. Samples were imaged at the equatorial plane to avoid photoselection. A pulsed titanium-sapphire (Mai-Tai Deepsee, Spectra-Physics, Santa Clara, CA, USA) laser tuned at 780 nm was used for two-photon imaging of Laurdan-labeled samples. Fluorescence emission was collected by non-descanned (NDD) hybrid detectors, as they offer a higher sensitivity compared to descanned photomultipliers. The blue edge of the emission spectrum was collected by NDD 1 at 435 ± 20 nm and the red edge by NDD 2 at 500 ± 10 nm. Irradiance at the sample plane was ≈500 GW·cm^−2^ for two-photon excitation [31].

### 3.5. Data and Image Analysis

A MATLAB (MathWorks, Natick, MA, USA)-based software was used to calculate GP values in cells, blebs, PM patches, and GUV images [32]. At least 150 images of each sample were analyzed. Images were smoothed in each channel with 2-pixel averaging (merging of surrounding 2 pixels) and the GP value was calculated using the following equation:

(1) $$GP=\frac{I_{B}-{G\;\times \;I}_{R}}{I_{B}+{G\;\times \;I}_{R}}$$

where *I*_B_ is the intensity collected by NDD 1, *I*_R_ is the intensity collected by NDD 2, and *G* is the correction factor [14]. The G factor is calculated by measuring the GP value of the same fluorophore concentration used in sample staining but dissolved in pure DMSO [15].

In the case of whole cell images, the region of interest (the PM) was manually selected to separate it from the rest of the cell when required.

### 3.6. Fluorescence Spectrometric Analysis

Whole cells, SUV formed from lipid extracts, GPMV (blebs) or PM patches were labeled with Laurdan. Whole cells, GPMV and PM patches were directly mixed at a final 82.5 µM lipid and 0.75 µM Laurdan concentrations. Lipid extracts in chloroform:methanol (2:1) were mixed with Laurdan and the solvent was evaporated to dryness under a stream of N_2_. Then, the sample was kept under vacuum for 2 h to remove solvent traces and the lipids were swollen in the appropriate buffer (NaCl 150mM, Hepes 25mM, pH 7.4) to a final concentration of 82.5 µM lipid and 0.75 µM Laurdan. Sonicated SUV were obtained with a probe-type Soniprep 150 sonicator (MSK, London, UK). Fluorescence measurements were performed using a QuantaMaster 40 spectrofluorometer (Photon Technology International, Lawrenceville, NJ, USA) [33].

### 3.7. AFM

GPMV, PM patches and supported planar bilayers (SPB) formed by cell lipid extracts were scanned by AFM. Cell lipid extract samples were prepared on V-2 high quality scratch-free mica substrates (Asheville-Schoonmaker Mica Co., Newport News, VA, USA). A total of 180 μL assay buffer containing 3 mM CaCl_2_ was added onto a 1.2 cm^2^ freshly cleaved mica substrate mounted onto a BioCell (JPK Instruments, Berlin, Germany). Then, 80 μL sonicated 0.4 mM SUV formed from CHO lipid extract were added on top of the mica slip. BioCell temperature was gradually increased (5 °C every 5 min) up to 80 °C. Vesicles were left to adsorb and extend for 30 min keeping the sample temperature at 80 °C. A further 30 min were allowed for the samples to equilibrate at room temperature before performing five washing steps with calcium-free buffer in order to discard non-adsorbed vesicles and remove the remaining Ca^2+^ cations [34].

#### 3.7.1. Blebs

Blebs were first stained using Di-4-ANEPPDHQ in order to know where the GPMV were exactly located on the mica slip. Then, samples were left for 3 h to sediment over the poly-lysine-coated mica slip.

#### 3.7.2. PM Patches

Isolated PM patches were prepared as described above, using poly-lysine-coated mica slips instead of glass-bottom dishes.

#### 3.7.3. Topographic Measurements

A NanoWizard II AFM (JPK Instruments, Berlin, Germany) was used to perform sample topographic measurements under contact mode scanning (constant vertical deflection). For a proper measurement, the AFM was coupled to a Leica microscope and mounted onto a Halcyonics Micro 40 antivibration table (Halcyonics, Inc., Menlo Park, CA, USA) and inside an acoustic enclosure (JPK Instruments). V-shaped MLCT Si_3_N_4_ cantilevers (Bruker, Billerica, MA, USA) with nominal spring constants 0.1 or 0.5 N/m were used for imaging. Sample thickness was estimated by cross-section height analysis. GPMV topographic measurements could not be performed because these structures would not flatten on the mica for AFM examination.

#### 3.7.4. Force Spectroscopy Measurements

V-shaped MLCT Si_3_N_4_ cantilevers (Bruker, Billerica, MA, USA) with nominal spring constants 0.1 or 0.5 N/m were individually calibrated with a lipid-free mica substrate in assay buffer using the thermal noise method. After proper bilayer area localization, force spectroscopy was performed at a speed of 1 μm/s. Force steps were determined for each of the indentation curves as reproducible jumps within the extended traces. At least three independent sample preparations were scanned for each case and 50–100 curves were measured in each of them.

### 3.8. Mass Spectroscopic Analysis

#### 3.8.1. Lipid Extraction

Lipid extraction was performed using a modified MTBE protocol [23]. Briefly, cells were washed with cold PBS and scraped off in 500 μL cold PBS on ice. The suspension was transferred to a 2 mL tube in which it was spun down at 3200 rpm for 5 min at 4 °C. After removing the PBS, samples were stored at −20 °C or directly used for further extraction. GPMV (blebs) and PM patch samples were prepared as previously mentioned. Then, 360 μL methanol was added and vortexed. A mixture of lipid standards (see Table 1) was added and samples were vortexed for 10 min at 4 °C using a Cell Disruptor Genie (Scientific Industries, Inc, city, state, contry). MTBE (1.2 mL) was then added and the samples were incubated for 1 h at room temperature with shaking (750 rpm). Phase separation was induced by adding 200 μL H_2_O. After 10 min incubation at room temperature, the sample was centrifuged at 1000 × g for 10 min. The upper (organic) phase was transferred to a 13 mm screw-cap glass tube and the lower phase was extracted with 400 μL artificial upper phase (MTBE/methanol/water (10:3:1.5, *v*/*v*/*v*)). The two upper phases were combined and the total lipid extract was divided in 3 equal aliquots (one for phospholipids (TL), one for sterols (S) in 2-mL amber vials, and one for sphingolipid (SL) detection in a 13-mm glass tube) and dried in a Centrivap at 50 °C or under a nitrogen flow. The SL aliquot was deacylated by methylamine treatment (Clarke method) to remove phospholipids. 0.5 mL monomethylamine reagent [MeOH/H_2_O/n-butanol/methylamine solution (4:3:1:5 *v*/*v*)] was added to the dried lipid, followed by sonication (5 min). Samples were then mixed and incubated for 1 h at 53 °C and dried (as above). The monomethylamine-treated lipids were desalted by n-butanol extraction. 300 μl H_2_O-saturated n-butanol was added to the dried lipids. The sample was vortexed, sonicated for 5 min, then 150 μl MS-grade water was added. The mixture was vortexed thoroughly and centrifuged at 3200 × g for 10 min. The upper phase was transferred to a 2-mL amber vial. The lower phase was extracted twice more with 300 μL H_2_O-saturated n-butanol and the upper phases were combined and dried as above.

#### 3.8.2. Glycerophospholipid and Sphingolipid Detection on a Triple Quadrupole Mass Spectrometer

TL and SL aliquots were resuspended in 250 μL chloroform/methanol (1:1 *v*/*v*) (LC-MS/HPLC grade) and sonicated for 5 min. The samples were pipetted in a 96-well plate (final volume = 100 μL). The TL were diluted 1:4 in negative-mode solvent (chloroform/methanol (1:2) + 5mM ammonium acetate) and 1:10 in positive-mode solvent (chloroform/methanol/water (2:7:1 *v*/*v*) + 5mM ammonium acetate). The SL were diluted 1:10 in positive-mode solvent and infused into the mass spectrometer. Tandem mass spectrometry for the identification and quantification of sphingolipid molecular species was performed using Multiple Reaction Monitoring (MRM) with a TSQ Vantage Triple Stage Quadrupole Mass Spectrometer (Thermo Fisher Scientific, Waltham, MA, USA) equipped with a robotic nanoflow ion source, Nanomate HD (Advion Biosciences, Ithaca, NY, USA). The collision energy was optimized for each lipid class. The detection conditions for each lipid class are listed below (Table 1). Ceramide species were also quantified with a loss of water in the first quadrupole. Each biological replica was read in 2 technical replicas. Each of these replicas comprised 3 measurements for each transition. Lipid concentrations were calculated relative to the relevant internal standards and then normalized to the total lipid content of each lipid extract (mol%).

### 3.9. Gas Chromatography–Mass Spectrometry for Cholesterol Assay

Lipid extracts were analyzed by GC-MS as described previously [35]. Briefly, samples were injected into a VARIAN CP-3800 gas chromatograph equipped with a FactorFour Capillary Column VF-5ms 15 m × 0.32 mm i.d. DF = 0.10, and analyzed in a Varian 320 MS triple quadrupole with electron energy set to −70 eV at 250 °C (Varian Agilent, Santa Clara, CA, USA). Samples were applied to the column oven at 45 °C, held for 4 min, then heated to 195 °C (20 °C/min). Sterols were eluted with a linear gradient from 195 to 230 °C (4 °C/min), followed by heating to 320 °C (10 °C/min). Cholesterol was identified by its retention time (compared with an ergosterol standard) and fragmentation patterns, which were compared with the NIST library.

## 4. Conclusions

Many significant plasma membrane studies at the molecular-cellular biophysical level are hampered by the lack of reliable preparative methods that will supply PM in sufficient amounts. In view of the interest of this subject we compared two different methods for the preparation of CHO cell plasma membranes, leading either to spherical GPMV (blebs) of flat patches. Both methods provide PM in sufficient amounts to allow its structural, biophysical, and biochemical study. Blebs and patches were found to be very similar, but not identical in composition or properties. Molecular lipid order and nanomechanical resistance are comparable for PM blebs and patches, but different (higher) than in the average cell membranes. Plasma membranes also exhibit peculiarities in their lipid composition, e.g. being rich in cholesterol and relatively poor in polyunsaturated fatty acids, as compared to the ensemble of cell membranes. These differences between PM preparations and whole cell membranes should not obscure the fact that other (minor, but significant) variations exist between blebs and patches, arising probably from the peculiar methods used in each preparation. The current work opens the way to more detailed structural and functional studies of CHO cell plasma membranes, and perhaps also cytoskeleton.

## Figures and Tables

**Table 1:** MS detection conditions for the different lipid classes.

Lipid Class	Standard	Polarity	Mode	m/z ion	Collision Energy
Phosphatidylcholine [M+H]^+^	DLPC	+	Product ion	184.07	30
Phosphatidylethanolamine [M+H]^+^	PE31:1	+	Neutral ion loss	141.02	20
Phosphatidylinositol [M-H]^−^	PI31:1	-	Product ion	241.01	44
Phosphatidylserine [M-H]^−^	PS31:1	-	Neutral ion loss	87.03	23
Cardiolipin [M-2H]^2−^	CL56:0	-	Product ion	acyl chain	32
Ceramide [M+H]^+^	C17Cer	+	Product ion	264.34	25
Dihydroceramide [M+H]^+^	C17Cer	+	Product ion	266.40	25
Hexosylceramide [M+H]^+^	C8GC	+	Product ion	264.34	30
Hexosyldihydroceramide [M+H]^+^	C8GC	+	Product ion	266.40	30
Sphingomyelin [M+H]^+^	C12SM	+	Product ion	184.07	26

## Supplementary Materials

Supplementary materials can be found at https://www.mdpi.com/1422-0067/21/7/2643/s1.
