# Supplementary material for: Patches and Blebs: A Comparative Study of the Composition and Biophysical Properties of Two Plasma Membrane Preparations from CHO Cells
Source: Int J Mol Sci. 2020 Apr 10;21(7):2643. doi: 10.3390/ijms21072643 (PMC7177368; doi:10.3390/ijms21072643)
Supplement: Supplementary file 1 [file ijms-21-02643-s001.pdf]

SUPPLEMENTARY MATERIAL

Patches and blebs: a comparative study of the composition and biophysical properties of two plasma membrane preparations from CHO cells

Bingen G. Monasterio [1,2], , Noemi Jiménez-Rojo [3], Aritz B. García-Arribas [1,2] Félix M. Goñi [1,2], Alicia Alonso [1,2*]

[1]Instituto Biofísika (CSIC, UPV/EHU) and [2]Departamento de Bioquímica, Universidad del País Vasco, Leioa, Spain; [3]NCCR Chemical Biology, Department of Biochemistry, University of Geneva, 1211 Geneva, Switzerland.

*Corresponding author. E-mail: alicia.alonso@ehu.eus

Table S1. A summary of the lipidomic data from whole CHO cells and derived plasma membrane preparations, blebs (or GPMV) and patches.

	GPMV control	SD GPMV control	GPMV	SD GPMV	PM patches control	PM patches control SD	PM patches	PM patches SD
**Total_CER_H2O**	0.27	0.06	0.76	0.11	0.41	0.00	0.45	0.03
**Total_GlcCER_H2O**	0.21	0.02	1.00	0.16	0.42	0.06	0.90	0.26
**Total_SM**	3.57	0.72	2.71	0.53	3.71	1.04	3.47	0.48
**Total_PC**	74.91	0.48	68.79	4.18	68.54	0.93	74.55	2.14
**Total_PE**	9.08	0.24	8.69	2.43	9.20	1.20	10.11	0.73
**Total_PI**	6.37	0.62	12.07	2.49	7.91	0.01	5.05	0.49
**Total_PS**	4.76	0.46	8.32	0.48	6.57	0.21	5.16	0.34
**Total_CL**	0.48	0.10	0.04	0.04	0.50	0.04	0.23	0.03
**Total_Chol**	3.28	0.19	23.39	1.76	3.12	0.17	5.91	0.29
**GPL_short**	10.30	0.21	25.79	1.91	8.10	0.12	12.00	0.58
**GPL_long**	84.63	0.77	68.98	1.55	82.66	0.96	82.46	1.31
**GPL_very_long**	0.46	0.02	0.44	0.11	0.50	0.02	0.41	0.03
**PC_Lyso**	0.08	0.00	0.27	0.02	0.14	0.01	0.05	0.01
**PE_Lyso**	0.07	0.00	0.13	0.04	0.07	0.01	0.03	0.00
**PI_Lyso**	0.04	0.00	0.21	0.08	0.09	0.01	0.05	0.01
**PS_Lyso**	0.01	0.00	0.46	0.23	0.01	0.00	0.02	0.01
**PC_Ether**	11.00	0.24	17.65	1.46	10.78	0.23	12.43	0.31
**PE_Ether**	0.23	0.01	4.75	2.17	0.23	0.00	0.47	0.15
**PI_Ether**	0.25	0.04	5.11	1.10	0.36	0.01	0.56	0.20
**PS_Ether**	0.15	0.01	1.06	0.38	0.28	0.01	0.21	0.02
**CER_H2O**	0.25	0.06	0.69	0.10	0.38	0.02	0.43	0.03
**DHCER_H2O**	0.02	0.01	0.05	0.01	0.02	0.00	0.01	0.00
**CERP_H2O**	0.00	0.00	0.02	0.00	0.01	0.00	0.00	0.00
**GlcCER_H2O**	0.21	0.02	0.98	0.16	0.42	0.06	0.89	0.27
**GlcDHCER_H2O**	0.00	0.00	0.02	0.00	0.00	0.00	0.01	0.00
**SM**	3.49	0.70	2.60	0.52	3.54	1.06	3.39	0.47
**DHSM**	0.08	0.01	0.11	0.01	0.34	0.02	0.08	0.01
**DB_0**	4.87	0.12	6.28	0.18	3.75	0.07	4.55	0.06
**DB_1**	41.31	0.94	33.65	3.76	37.39	0.33	40.96	0.58
**DB_2**	23.42	0.05	14.12	1.71	23.62	0.44	24.99	0.88
**DB_3**	3.41	0.15	2.29	0.36	3.31	0.06	2.92	0.12
**DB_4**	4.70	0.21	1.79	0.15	4.96	0.03	3.30	0.07
**DB_5**	3.39	0.19	2.14	0.19	3.77	0.07	2.42	0.09
**DB_6**	2.46	0.17	5.29	1.34	2.48	0.04	1.92	0.12
**CL_2_DB**	0.06	0.06	0.00	0.00	0.00	0.00	0.00	0.00
**CL_3_DB**	0.04	0.00	0.00	0.00	0.04	0.00	0.02	0.00
**CL_4_DB**	0.11	0.02	0.00	0.00	0.13	0.02	0.07	0.01

**CL_5_DB**	0.13	0.01	0.00	0.01	0.20	0.01	0.09	0.01
**CL_6_DB**	0.10	0.01	0.02	0.01	0.10	0.01	0.04	0.00
**CL_7_DB**	0.02	0.00	0.00	0.00	0.02	0.00	0.01	0.00
**PC_UI_0**	5.38	0.13	7.47	0.12	4.09	0.05	5.15	0.10
**PC_UI_1**	40.08	0.84	42.47	2.02	35.24	0.58	40.56	0.62
**PC_UI_2**	21.89	0.19	14.00	1.73	21.89	0.47	23.33	1.27
**PC_UI_3**	2.68	0.12	1.74	0.24	2.55	0.07	2.31	0.18
**PC_UI_4**	2.01	0.13	1.18	0.15	1.85	0.02	1.28	0.11
**PC_UI_5**	1.65	0.13	0.95	0.13	1.74	0.02	1.07	0.09
**PC_UI_6**	1.22	0.12	0.97	0.08	1.21	0.02	0.84	0.06
**PE_UI_0**	0.21	0.01	0.43	0.07	0.21	0.01	0.26	0.03
**PE_UI_1**	2.23	0.05	1.69	0.20	1.89	0.02	2.96	0.24
**PE_UI_2**	2.20	0.05	1.13	0.21	1.92	0.05	2.81	0.19
**PE_UI_3**	0.50	0.03	0.53	0.16	0.40	0.01	0.56	0.05
**PE_UI_4**	1.23	0.04	3.12	1.16	1.10	0.01	1.32	0.17
**PE_UI_5**	1.42	0.07	0.39	0.08	1.39	0.04	1.21	0.06
**PE_UI_6**	1.29	0.07	0.39	0.06	1.19	0.03	1.00	0.01
**PI_UI_0**	0.16	0.02	0.28	0.03	0.24	0.00	0.12	0.00
**PI_UI_1**	1.79	0.32	1.13	0.34	2.16	0.07	1.20	0.05
**PI_UI_2**	1.43	0.15	3.65	0.21	1.75	0.04	1.46	0.21
**PI_UI_3**	0.63	0.04	0.66	0.08	0.74	0.01	0.50	0.04
**PI_UI_4**	1.81	0.09	0.43	0.08	2.28	0.07	1.21	0.04
**PI_UI_5**	0.41	0.02	1.10	0.30	0.57	0.02	0.30	0.06
**PI_UI_6**	0.14	0.01	2.24	0.49	0.17	0.01	0.26	0.11
**PS_UI_0**	0.30	0.02	3.05	0.06	0.31	0.01	0.21	0.00
**PS_UI_1**	2.56	0.29	2.63	0.16	3.80	0.09	3.20	0.27
**PS_UI_2**	0.40	0.04	0.27	0.03	0.57	0.02	0.52	0.02
**PS_UI_3**	0.18	0.01	0.54	0.34	0.20	0.01	0.18	0.02
**PS_UI_4**	0.39	0.04	1.7	0.06	0.42	0.02	0.25	0.01
**PS_UI_5**	0.70	0.05	0.48	0.09	0.84	0.06	0.47	0.03
**PS_UI_6**	0.43	0.03	0.56	0.10	0.48	0.02	0.33	0.01
**PC_short**	10.03	0.22	22.12	1.34	7.78	0.13	11.56	0.48
**PC_long**	64.71	0.52	46.53	5.30	60.37	0.96	62.85	2.60
**PC_very_long**	0.17	0.01	0.14	0.03	0.16	0.01	0.14	0.03
**PE_short**	0.15	0.01	1.24	0.23	0.15	0.00	0.23	0.04
**PE_long**	8.85	0.24	7.45	2.21	7.93	0.13	9.81	0.69
**PE_very_long**	0.08	0.00	0.00	0.00	0.07	0.00	0.07	0.00
**PI_short**	0.10	0.01	0.77	0.05	0.09	0.00	0.11	0.02
**PI_long**	6.16	0.59	11.02	2.31	7.73	0.01	4.84	0.46
**PI_very_long**	0.11	0.01	0.28	0.16	0.09	0.00	0.10	0.00
**PS_short**	0.16	0.00	3.77	0.46	0.08	0.00	0.11	0.04
**PS_long**	4.68	0.44	5.40	0.17	6.29	0.20	4.96	0.31
**PS_very_long**	0.11	0.02	0.05	0.02	0.19	0.01	0.09	0.01
**CER_H2O_10**	0.00	0.00	0.01	0.00	0.00	0.00	0.00	0.00

**CER_H2O_14**	0.00	0.00	0.01	0.01	0.00	0.00	0.00	0.00
**CER_H2O_16**	0.13	0.03	0.21	0.02	0.19	0.01	0.27	0.01
**CER_H2O_18**	0.01	0.00	0.09	0.03	0.01	0.00	0.01	0.00
**CER_H2O_20**	0.00	0.00	0.04	0.01	0.00	0.00	0.00	0.00
**CER_H2O_22**	0.01	0.00	0.05	0.01	0.06	0.01	0.02	0.00
**CER_H2O_24**	0.11	0.03	0.25	0.03	0.16	0.01	0.14	0.02
**CER_H2O_26**	0.00	0.00	0.10	0.03	0.00	0.00	0.01	0.00
**GlcCER_H2O_10**	0.00	0.00	0.03	0.02	0.00	0.00	0.00	0.00
**GlcCER_H2O_12**	0.00	0.00	0.02	0.01	0.01	0.00	0.00	0.00
**GlcCER_H2O_14**	0.00	0.00	0.03	0.02	0.00	0.00	0.00	0.00
**GlcCER_H2O_16**	0.13	0.01	0.61	0.10	0.27	0.04	0.60	0.16
**GlcCER_H2O_18**	0.00	0.00	0.03	0.00	0.00	0.00	0.01	0.00
**GlcCER_H2O_20**	0.00	0.00	0.01	0.01	0.00	0.00	0.00	0.00
**GlcCER_H2O_22**	0.01	0.00	0.05	0.00	0.02	0.01	0.05	0.02
**GlcCER_H2O_24**	0.07	0.01	0.20	0.05	0.12	0.01	0.23	0.08
**GlcCER_H2O_26**	0.00	0.00	0.01	0.00	0.00	0.00	0.00	0.00
**SM_14**	0.03	0.00	0.03	0.00	0.05	0.01	0.03	0.00
**SM_16**	3.23	0.70	2.45	0.48	5.26	1.22	3.23	0.43
**SM_18**	0.01	0.01	0.01	0.00	0.12	0.02	0.01	0.00
**SM_20**	0.00	0.00	0.00	0.00	0.12	0.03	0.00	0.00
**SM_22**	0.03	0.00	0.06	0.00	0.52	0.12	0.02	0.00
**SM_24**	0.27	0.03	0.16	0.04	0.53	0.05	0.18	0.05
**PC_28**	0.14	0.02	0.42	0.04	0.07	0.00	0.16	0.02
**PC_30**	1.83	0.07	4.97	0.40	1.10	0.08	2.94	0.37
**PC_32**	8.03	0.14	16.65	1.55	6.61	0.07	8.44	0.09
**PC_34**	35.53	1.26	26.39	3.00	30.83	0.55	33.28	0.99
**PC_36**	23.35	0.45	15.67	1.90	23.82	0.40	24.46	1.40
**PC_38**	4.78	0.34	3.64	0.53	4.67	0.05	4.36	0.21
**PC_40**	1.00	0.08	0.64	0.06	1.00	0.02	0.71	0.07
**PC_42**	0.14	0.01	0.10	0.02	0.13	0.01	0.11	0.02
**PC_44**	0.03	0.00	0.04	0.01	0.03	0.00	0.03	0.01
**PE_28**	0.00	0.00	0.04	0.01	0.00	0.00	0.00	0.00
**PE_30**	0.01	0.00	0.44	0.10	0.01	0.00	0.03	0.01
**PE_32**	0.13	0.00	0.69	0.10	0.13	0.00	0.19	0.03
**PE_34**	1.34	0.04	4.75	1.96	1.12	0.02	1.84	0.21
**PE_36**	3.38	0.05	1.73	0.39	3.00	0.05	4.40	0.35
**PE_38**	1.92	0.10	0.52	0.14	1.76	0.02	1.83	0.09
**PE_40**	2.15	0.10	0.39	0.13	1.96	0.06	1.71	0.07
**PE_42**	0.07	0.00	0.00	0.00	0.05	0.00	0.06	0.00
**PE_44**	0.01	0.00	0.00	0.00	0.01	0.00	0.01	0.00
**PI_28**	0.00	0.00	0.03	0.03	0.00	0.00	0.00	0.00
**PI_30**	0.02	0.00	0.25	0.08	0.01	0.00	0.03	0.01
**PI_32**	0.07	0.01	0.33	0.06	0.08	0.00	0.07	0.01
**PI_34**	0.94	0.17	0.28	0.18	1.16	0.02	0.54	0.04

**PI_36**	2.28	0.31	4.23	1.12	2.89	0.10	1.90	0.21
**PI_38**	2.44	0.12	5.06	0.86	3.04	0.09	2.04	0.24
**PI_40**	0.46	0.03	1.40	0.48	0.55	0.02	0.33	0.06
**PI_42**	0.08	0.01	0.12	0.09	0.06	0.00	0.07	0.00
**PI_44**	0.03	0.00	0.16	0.10	0.02	0.00	0.03	0.00
**PS_30**	0.00	0.00	0.35	0.07	0.00	0.00	0.02	0.02
**PS_32**	0.02	0.00	0.84	0.17	0.08	0.00	0.06	0.02
**PS_34**	0.52	0.05	1.12	0.27	0.56	0.02	0.53	0.02
**PS_36**	2.77	0.26	1.82	0.18	3.60	0.08	3.06	0.24
**PS_38**	0.36	0.03	0.20	0.03	0.46	0.03	0.35	0.01
**PS_40**	1.24	0.12	0.84	0.11	1.68	0.09	1.01	0.05
**PS_42**	0.09	0.02	0.01	0.02	0.18	0.01	0.09	0.01
**PS_44**	0.01	0.00	0.00	0.00	0.01	0.00	0.00	0.00
**CL_68**	0.05	0.01	0.00	0.00	0.06	0.01	0.03	0.00
**CL_70**	0.23	0.07	0.00	0.00	0.21	0.02	0.11	0.02
**CL_72**	0.17	0.01	0.00	0.00	0.21	0.02	0.09	0.01
**CL_74**	0.02	0.01	0.02	0.01	0.02	0.00	0.01	0.00
**CL_76**	0.00	0.00	0.02	0.03	0.00	0.00	0.00	0.00
**PC_Lyso_short**	0.03	0.00	0.09	0.01	0.07	0.01	0.02	0.00
**PC_Lyso_long**	0.05	0.00	0.19	0.02	0.07	0.00	0.03	0.01
**PE_Lyso_short**	0.01	0.00	0.07	0.04	0.01	0.00	0.00	0.00
**PE_Lyso_long**	0.06	0.00	0.06	0.03	0.06	0.01	0.03	0.00
**PI_Lyso_short**	0.00	0.00	0.16	0.08	0.01	0.00	0.01	0.00
**PI_Lyso_long**	0.04	0.00	0.05	0.03	0.08	0.01	0.04	0.01
**PS_Lyso_short**	0.00	0.00	0.46	0.23	0.00	0.00	0.02	0.01
**PC_Lyso_UI_0**	0.05	0.00	0.20	0.01	0.09	0.00	0.03	0.00
**PC_Lyso_UI_1**	0.04	0.00	0.04	0.01	0.05	0.00	0.02	0.00
**PC_Lyso_UI_2**	0.00	0.00	0.04	0.01	0.00	0.00	0.00	0.00
**PE_Lyso_UI_0**	0.03	0.00	0.01	0.01	0.03	0.00	0.01	0.00
**PE_Lyso_UI_1**	0.04	0.00	0.09	0.05	0.04	0.01	0.02	0.00
**PE_Lyso_UI_2**	0.00	0.00	0.02	0.01	0.00	0.00	0.00	0.00
**PI_Lyso_UI_0**	0.03	0.00	0.05	0.03	0.07	0.01	0.03	0.01
**PI_Lyso_UI_1**	0.01	0.00	0.16	0.08	0.02	0.00	0.01	0.00
**PS_Lyso_UI_1**	0.00	0.00	0.46	0.23	0.00	0.00	0.02	0.01
**PC_Ether_short**	1.10	0.03	10.38	2.01	0.90	0.04	1.80	0.27
**PC_Ether_long**	9.83	0.22	7.22	0.74	9.80	0.24	10.57	0.54
**PC_Ether_very_long**	0.07	0.00	0.05	0.01	0.07	0.01	0.06	0.02
**PE_Ether_short**	0.00	0.00	0.32	0.06	0.02	0.00	0.02	0.01
**PE_Ether_long**	0.23	0.01	4.43	2.12	0.21	0.00	0.45	0.14
**PI_Ether_short**	0.01	0.00	0.14	0.03	0.02	0.00	0.01	0.01
**PI_Ether_long**	0.23	0.04	4.86	1.04	0.33	0.01	0.53	0.19
**PI_Ether_very_long**	0.01	0.00	0.11	0.09	0.01	0.00	0.01	0.00
**PS_Ether_short**	0.00	0.00	0.11	0.03	0.06	0.00	0.01	0.01
**PS_Ether_long**	0.14	0.01	0.95	0.36	0.20	0.02	0.19	0.02

**PS_Ether_very_long**	0.01	0.00	0.00	0.00	0.01	0.00	0.00	0.00
**PC_Ether_UI_0**	0.95	0.04	1.65	0.02	0.79	0.01	1.09	0.03
**PC_Ether_UI_1**	5.45	0.15	13.20	1.58	5.47	0.17	6.63	0.12
**PC_Ether_UI_2**	2.39	0.09	1.50	0.19	2.47	0.10	2.68	0.19
**PC_Ether_UI_3**	0.52	0.02	0.31	0.04	0.52	0.02	0.53	0.07
**PC_Ether_UI_4**	0.61	0.02	0.31	0.05	0.58	0.01	0.53	0.07
**PC_Ether_UI_5**	0.61	0.03	0.31	0.05	0.66	0.02	0.53	0.05
**PC_Ether_UI_6**	0.48	0.03	0.37	0.04	0.47	0.01	0.44	0.04
**PE_Ether_UI_0**	0.00	0.00	0.17	0.04	0.02	0.00	0.01	0.00
**PE_Ether_UI_1**	0.04	0.00	0.19	0.02	0.03	0.00	0.06	0.01
**PE_Ether_UI_2**	0.01	0.00	0.13	0.04	0.01	0.00	0.03	0.01
**PE_Ether_UI_3**	0.01	0.00	0.28	0.10	0.01	0.00	0.04	0.01
**PE_Ether_UI_4**	0.04	0.00	3.86	1.95	0.04	0.00	0.21	0.12
**PE_Ether_UI_5**	0.04	0.00	0.11	0.05	0.04	0.00	0.06	0.01
**PE_Ether_UI_6**	0.07	0.00	0.01	0.01	0.07	0.00	0.07	0.00
**PI_Ether_UI_0**	0.00	0.00	0.05	0.01	0.03	0.00	0.00	0.00
**PI_Ether_UI_1**	0.06	0.01	0.33	0.09	0.09	0.00	0.07	0.01
**PI_Ether_UI_2**	0.10	0.01	4.33	1.02	0.11	0.00	0.40	0.17
**PI_Ether_UI_3**	0.03	0.01	0.11	0.04	0.04	0.00	0.03	0.01
**PI_Ether_UI_4**	0.03	0.00	0.03	0.01	0.05	0.00	0.02	0.00
**PI_Ether_UI_5**	0.02	0.00	0.21	0.07	0.04	0.00	0.03	0.01
**PI_Ether_UI_6**	0.01	0.00	0.04	0.01	0.00	0.00	0.01	0.00
**PS_Ether_UI_0**	0.01	0.00	0.08	0.02	0.07	0.00	0.01	0.00
**PS_Ether_UI_1**	0.09	0.01	0.17	0.02	0.13	0.01	0.12	0.01
**PS_Ether_UI_2**	0.02	0.00	0.03	0.02	0.03	0.00	0.02	0.00
**PS_Ether_UI_3**	0.00	0.00	0.76	0.35	0.00	0.00	0.04	0.01
**PS_Ether_UI_5**	0.01	0.00	0.01	0.00	0.02	0.00	0.01	0.00
**PS_Ether_UI_6**	0.01	0.00	0.02	0.01	0.01	0.00	0.01	0.00
**PC_Ether_30**	0.15	0.01	0.35	0.01	0.09	0.00	0.18	0.01
**PC_Ether_32**	0.95	0.04	10.04	2.00	0.81	0.04	1.62	0.26
**PC_Ether_34**	4.66	0.19	3.76	0.41	4.72	0.22	5.27	0.12
**PC_Ether_36**	3.35	0.10	2.36	0.20	3.49	0.12	3.53	0.28
**PC_Ether_38**	1.46	0.08	0.91	0.16	1.49	0.04	1.46	0.11
**PC_Ether_40**	0.36	0.02	0.20	0.01	0.37	0.01	0.31	0.05
**PC_Ether_42**	0.06	0.00	0.04	0.01	0.06	0.00	0.05	0.02
**PC_Ether_44**	0.01	0.00	0.01	0.00	0.01	0.00	0.01	0.00
**PE_Ether_30**	0.00	0.00	0.21	0.03	0.00	0.00	0.01	0.00
**PE_Ether_32**	0.00	0.00	0.11	0.03	0.02	0.00	0.01	0.00
**PE_Ether_34**	0.07	0.00	4.13	2.01	0.05	0.00	0.25	0.13
**PE_Ether_36**	0.03	0.00	0.16	0.06	0.03	0.00	0.06	0.01
**PE_Ether_38**	0.09	0.00	0.13	0.06	0.09	0.00	0.10	0.01
**PE_Ether_40**	0.04	0.00	0.00	0.01	0.04	0.00	0.03	0.00
**PI_Ether_30**	0.00	0.00	0.11	0.03	0.00	0.00	0.01	0.01
**PI_Ether_32**	0.00	0.00	0.03	0.00	0.02	0.00	0.00	0.00

**PI_Ether_34**	0.00	0.00	0.04	0.01	0.00	0.00	0.00	0.00
**PI_Ether_36**	0.08	0.02	0.13	0.02	0.14	0.00	0.07	0.00
**PI_Ether_38**	0.11	0.02	4.69	1.02	0.14	0.01	0.45	0.19
**PI_Ether_40**	0.03	0.00	0.00	0.00	0.04	0.00	0.01	0.00
**PI_Ether_42**	0.01	0.00	0.11	0.09	0.01	0.00	0.01	0.00
**PS_Ether_30**	0.00	0.00	0.09	0.02	0.00	0.00	0.01	0.00
**PS_Ether_32**	0.00	0.00	0.02	0.01	0.06	0.01	0.00	0.00
**PS_Ether_34**	0.01	0.00	0.86	0.37	0.02	0.00	0.06	0.01
**PS_Ether_36**	0.07	0.01	0.06	0.02	0.11	0.01	0.09	0.00
**PS_Ether_38**	0.03	0.00	0.03	0.01	0.05	0.01	0.04	0.00
**PS_Ether_40**	0.02	0.00	0.00	0.00	0.02	0.00	0.01	0.00

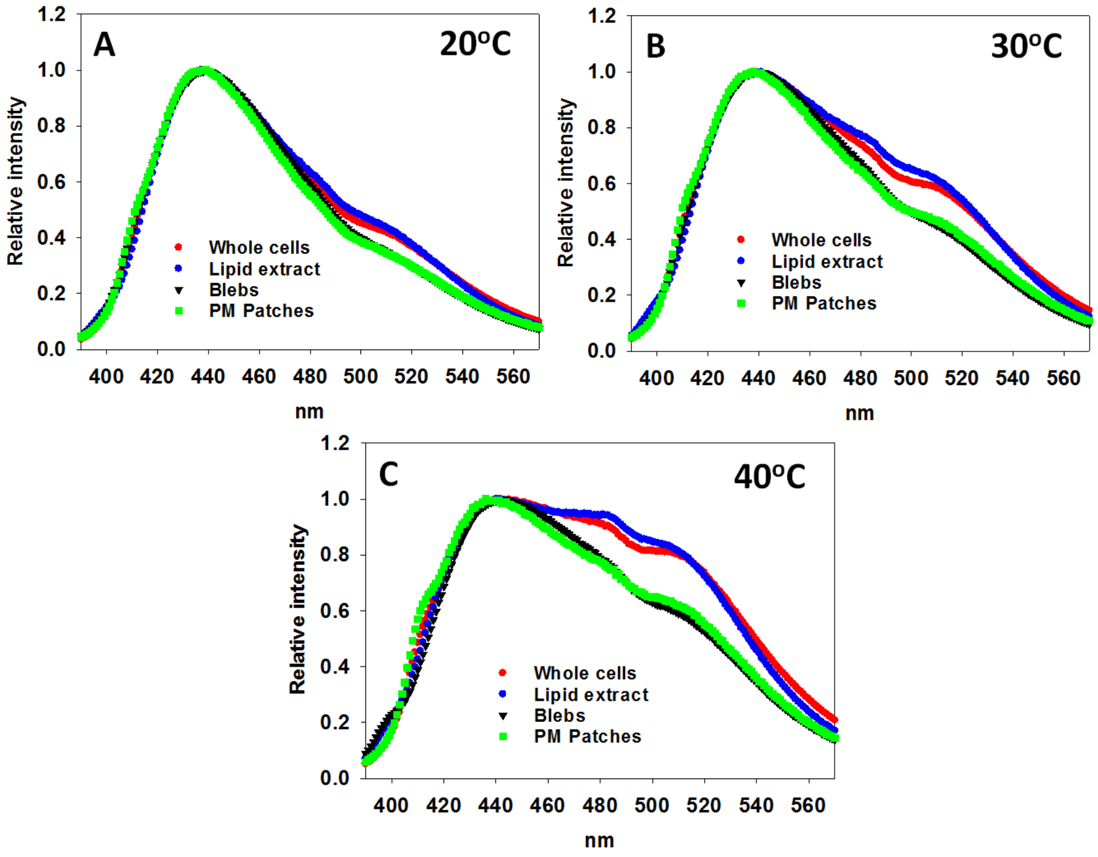

Figure S1. Laurdan GP measurements. Phase fluctuation in phospholipid membranes revealed by Laurdan fluorescence at (**A**) 20°C, (**B**) 30°C, and (**C**) 40°C. Red, whole CHO cells; blue, SUV formed from CHO cell lipid extract; black, blebs from CHO cells; green, PM patches from CHO cells.

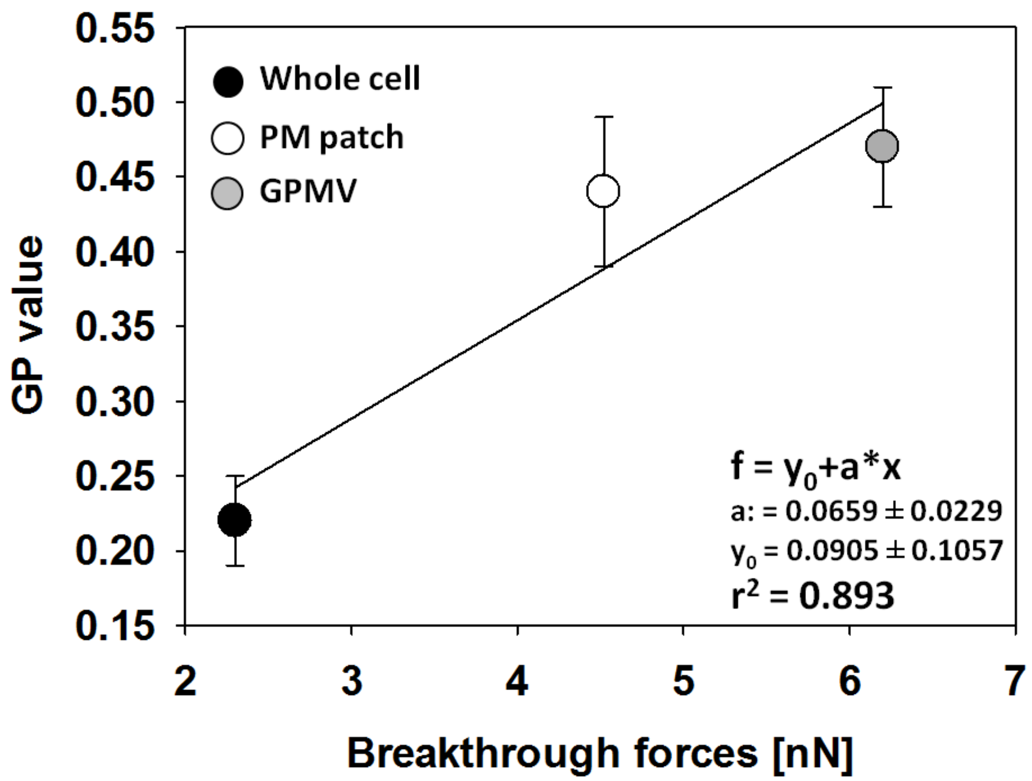

Figure S2. AFM - Laurdan fluorescence correlation. The correlation between Laurdan GP and bilayer breakthrough forces is shown as a regression line (r^2 = 0.893). The experimental points correspond to whole cells (black), PM patches (white), and GPMV (gray circle). Average values ± S.D.

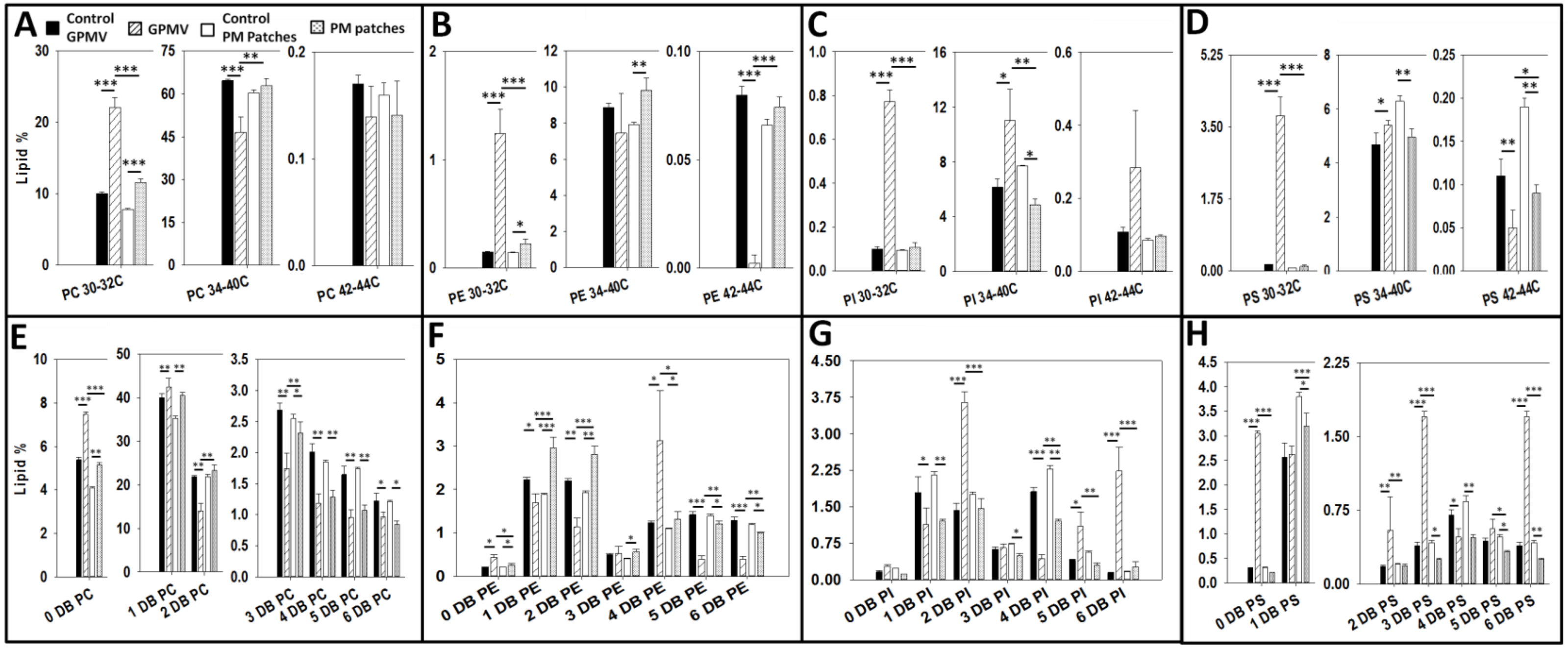

Figure S3. Lipid distribution according to chain length and saturation level (DB = double bond) of A and E, PC; B and F, PE; C and G, PI and D and H, PS. Bars: solid black, whole cells treated for GPMV preparation; striped, GPMV (blebs); empty, cells treated for PM patch preparation; dotted PM patches. Significance: (*) $p < 0.05$; (**0 $p < 0.01$; (***) $p < 0.001$

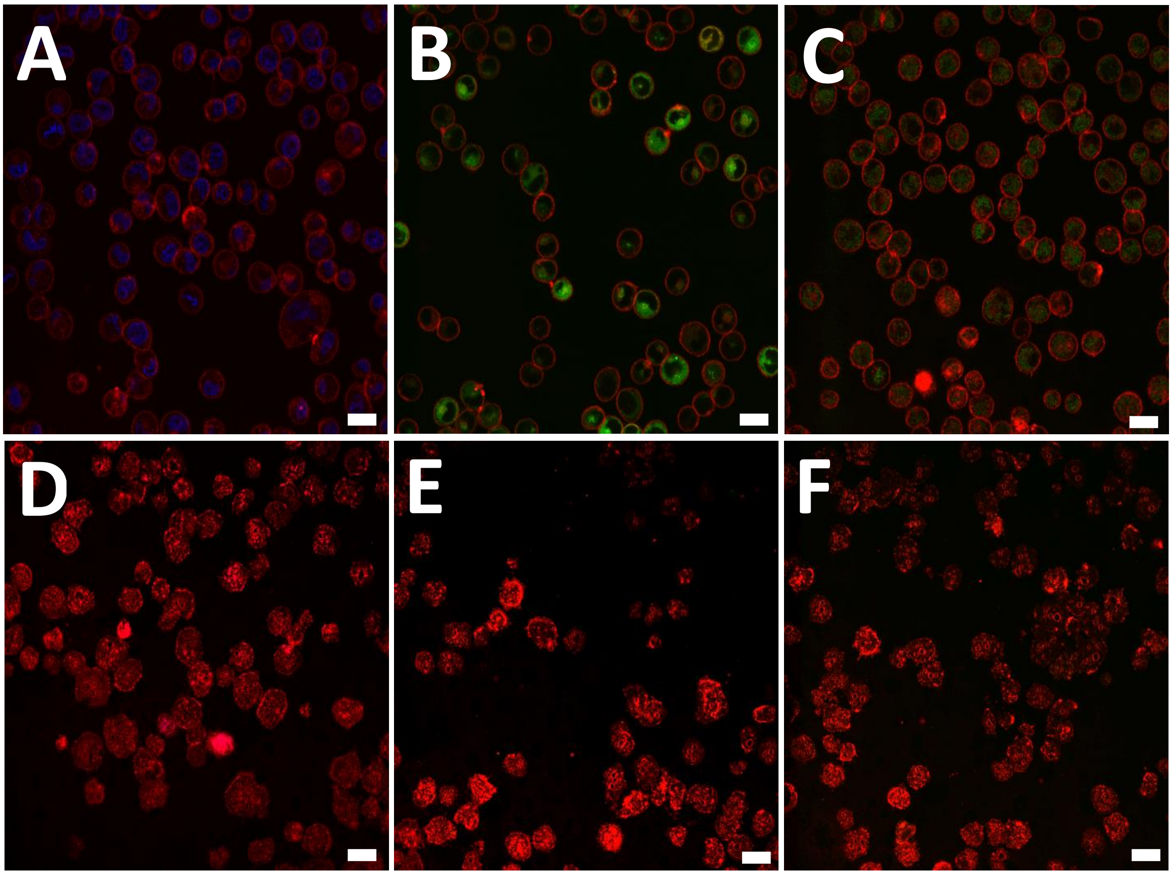

Figure S4. PM patch purification. (**A-C**), whole cells; (**D-F**), PM patches. All samples have been treated with Di-4 ANEPPDHQ as a general membrane fluorescent stain. In addition, the following organelle-specific dyes have been used: Hoechst 33342 for nuclei (**A** and **D**), Mitotracker Green for mitochondria (**B** and **E**), Bodipy-FL-C5-Cer for Golgi (**C** and **F**). Bar 20 µm
